# A Pilot Survey Study of Adherence to Care Considerations for Duchenne Muscular Dystrophy

**DOI:** 10.1371/currents.md.5f533e6e60ee172d6bf6b2b8375dfadf

**Published:** 2018-05-11

**Authors:** Kristin Conway, Christina Trout, Christina Westfield, Deborah Fox, Shree Pandya

**Affiliations:** Department of Epidemiology, University of Iowa, Iowa City, IA, USA; Stead Family Department of Pediatrics, University of Iowa, Iowa City, IA, USA; Bureau of Environmental and Occupational Epidemiology, New York State Department of Health, Buffalo, New York, USA; Department of Malformations Registry, New York State Department of Health, Albany, New York, USA; Department of Neurology, University of Rochester, Rochester, New York, USA

## Abstract

Introduction Care Considerations supported by the Centers for Disease Control and Prevention for the management of Duchenne muscular dystrophy were published in 2010, but there has been limited study of implementation in the United States. Methods A questionnaire collecting information about standard care practices and perceived barriers was piloted by 9 clinic directors of facilities within the Muscular Dystrophy Surveillance, Tracking and Research network. Results Six clinic directors completed the questionnaire; 1 adult-only clinic was excluded. Over 80% adherence was found for 30 of 55 recommendations examined. Greatest variability was for initiation of corticosteroids, bone health monitoring, type of pulmonary function testing, and psychosocial management. Barriers included unclear guidelines, inadequate time and funding, family-specific barriers and lack of empirical support for some recommendations. Discussion This pilot study showed implementation of the 2010 Care Considerations, except for recommendations based largely on expert consensus. Complete adherence requires more studies and active promotion.

## A pilot survey study of adherence to care considerations for Duchenne muscular dystrophy

**Introduction**

Duchenne muscular dystrophy (DMD) is an X-linked genetic disorder of the dystrophin (DMD) gene and is well known as a complex, disabling disorder with shortened life expectancy. Advances in the clinical management of DMD have resulted in prolonged survival.^1^^, ^2^, ^3 In order to improve the consistency of treatment among providers, consensus-based Care Considerations for comprehensive, multi-disciplinary clinical management of patients with DMD were developed by an international panel of experts convened and supported by the C^3^enters for Disease Control and Prevention (CDC).^4^^, ^5

Survey studies on the provision of care to patients with DMD have been conducted using questionnaire responses from selected medical specialties^6^^, ^7 and patients or care providers.^8^^, ^9 In 2013, the CDC funded a pilot study to evaluate implementation of the 2010 Duchenne care recommendations in the United States. A questionnaire was designed to collect information about standard DMD care practices in clinics specializing in the treatment of children with DMD and the perceived barriers to provision of recommended care. The questionnaire was sent to clinic directors of Muscular Dystrophy Association (MDA) supported facilities located within the Muscular Dystrophy Surveillance, Tracking, and Research network (MD STARnet), a population-based surveillance program established in 2004 by the CDC.^10^^, ^11 In this paper, we report the findings from the pilot study, compare reported clinical practices to those delineated in the Care Considerations, and describe barriers to care as perceived by the directors.

**Methods**

**Questionnaire Development**

The pilot questionnaire was developed from existing data items in several programs, including the Canadian Paediatric Neuromuscular Physicians Survey,^6^ the Muscular Dystrophy Association (MDA) DMD registry items, Parent Project Muscular Dystrophy (PPMD) association’s metrics for certified Duchenne care clinics,^12^ and the Care-NMD Patient Survey (http://en.care-nmd.eu/). After compiling existing data items, the research team developed additional questions or added response categories not already included. In addition, a literature review of challenges to translating clinical guidelines into practice was conducted to identify potential barriers that may influence implementation.^13^^, ^14 Based on the review, specific questions regarding perceived provider, patient/family and system barriers to implementation were added to the questionnaire.

The questionnaire was reviewed by representatives of two patient support organizations (JW for MDA and KK for PPMD) for content, breadth, and interpretability. It was piloted by a neuromuscular physician and a nurse practitioner who were involved in DMD care but not associated with the research project. The final questionnaire consisted of 30 multiple-choice questions and four open-ended questions asking about opinions regarding future recommendations, areas of disagreement with current recommendations, and suggestions to improve implementation.

For reference, the questionnaire can be found at the following link: https://osf.io/65us2.

**Questionnaire Implementation**

A paper questionnaire was mailed to nine directors of MDA supported clinics located in participating MD STARnet sites (Arizona, Colorado, Iowa, western New York) in the fall of 2014. The questionnaire was expected to take 30 minutes to complete. Reminder emails and letters were sent at two and four weeks after the initial email. Responses were entered into a Research Electronic Data Capture (REDCap) database, a secure web application for managing online questionnaires and databases, housed at the University of Iowa. IRB approval was received by the New York State Department of Health Institutional Review Board (IRB number: 03-062/15-0643/15-0644).

**Statistical Analyses**

Percent adherence was calculated by combining response categories that met the minimum recommendation and dividing by the total number of responding clinic directors. SAS® software, Version 9.4 was used for analyses [Copyright (c) 2002-2012 by SAS Institute Inc., Cary, NC, USA.]. Responses to individual questions are available from the first author.

**Results**

Questionnaires were completed by six clinic directors: two from pediatric clinics, three from mixed pediatric and adult clinics, and one adult only clinic. Questionnaire responses from the adult only clinic director were excluded since the responses pertained only to care of adult patients and the focus of our study was the care provided from the time of diagnosis. Percentages for adherence across all remaining clinics are presented in Table 1. Genetic testing and counseling services were reported as provided by all clinics (Table 1 – Diagnostic Assessment). Access to a multi-disciplinary team of medical providers was reported as available by all but one clinic director (Table 1 – Disease Management Team; for a list of specialties, see Clinic Director Questionnaire in Supplementary Materials). Specialties reported as not available by at least one clinic included: pulmonology; gastroenterology; psychiatry or psychology; physical, occupational or speech therapy; and palliative care clinic or services (data not shown). All clinic directors reported completing at least one recommended assessment every 6 months with variability across the types of assessments completed (Table 1 – Clinical Assessment). Recommended clinical management also varied; all clinic directors recommended stretching, bracing for positioning or standing, adaptive/assistive equipment and wheelchairs, but fewer recommended wrist/hand splints, bracing for walking and communication devices.

**Table 1. Clinic director reports of care provided and adherence to recommended care.**
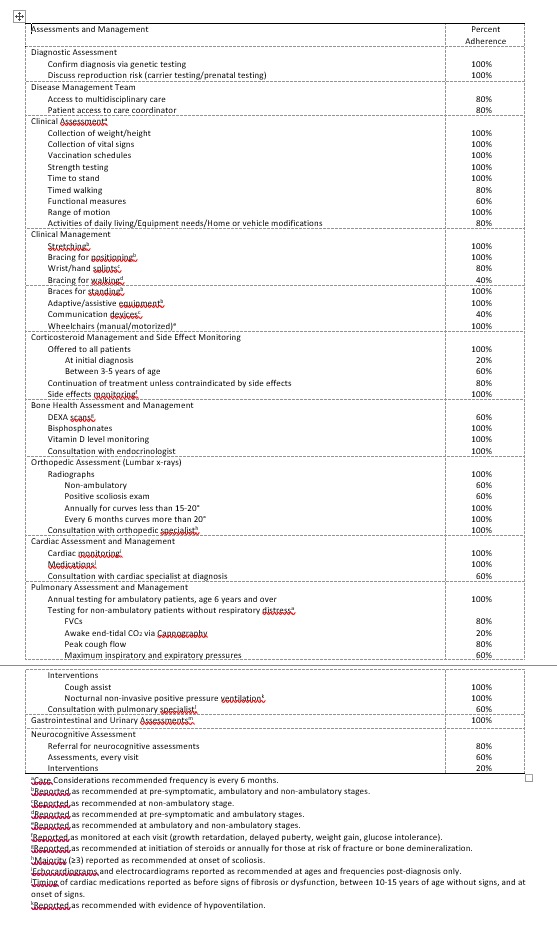

All clinic directors reported practice patterns consistent with the recommendations, including continued use of corticosteroids unless contraindicated (Table 1 – Corticosteroid Management and Side Effect Monitoring), but showed some variability in recommended age at initiation. Monitoring of side effects occurred at the recommended frequency. Variable adherence was found for the assessment of bone health (Table 1 – Bone Health Assessment and Management). Of those who assessed bone health, testing was completed at baseline and annually for high-risk patients (data not shown). Conversely, all directors reported offering bone health interventions consistent with recommended care (vitamin D, bisphosphonates) and consultations with endocrinologists to assess and treat osteopenia/osteoporosis. All clinic directors reported the use of radiographs to monitor scoliosis with variability in the indications for completing (Table 1 – Orthopedic Assessment).

Directors from all clinics reported recommending the use of electrocardiograms and echocardiograms for cardiac monitoring (Table 1 – Cardiac Assessment and Management); cardiac MRIs were not reported by any directors (data not shown). Adherence to frequency of monitoring was variable for patients under the age of 10, but consistent with the Care Considerations for those 10 and over. Holter monitoring was reported as recommended only if the child was symptomatic by all directors (data not shown). Medications were reported as used as treatment of cardiac dysfunction by all clinic directors. ACE inhibitor-angiotensin-converting-enzyme inhibitor was reported as the first medication recommended by all directors, but timing of use varied. Although all clinic directors reported referrals to a cardiac specialist, the timing of referrals varied (at diagnosis, at age 10, or abnormal cardiac test) (data not shown).

Annual monitoring of respiratory function of ambulatory boys ages 6 years and older was reported as recommended by all clinic directors (Table 1 – Pulmonary Assessment and Management). For non-ambulatory boys without respiratory symptoms or use of assisted ventilation, forced vital capacity and peak cough flow measures were reported as occurring at the recommended frequency by the majority (≥3) directors (Table 1). Of those not meeting the recommended frequency, pulmonary testing was reported as not being measured or tested annually (data not shown). The remaining pulmonary tests were reported less frequently as having been measured. Sleep studies were recommended with signs and symptoms and screening for awake or sleep hypoventilation every 6 months by all directors (data not shown). For the management of pulmonary dysfunction, cough assist and non-invasive ventilation were recommended by all clinic directors under multiple respiratory conditions (Table 1). Referrals to pulmonary specialists were not reported as recommended by all clinic directors, and the reasons for referral varied.

All clinic directors reported regular monitoring of gastrointestinal and urinary morbidities (Table 1 – Gastrointestinal and Urinary Assessments) and consultation with a nutritionist. The timing of the consultation typically occurred at initiation of steroids, after excessive weight gain, onset of chronic constipation, or when there was evidence of dysphagia (data not shown).

Only one clinic reported not completing a formal neuropsychological assessment before entering school (Table 1 – Neurocognitive Assessment); the remaining clinic directors reported either always completing an evaluation or completing if clinically indicated (data not shown). Evaluating for problems with coping, speech and language, learning disability, neurobehavioral disorders, emotional problems, or signs of social isolation at each visit were reported by most clinic directors. Psychotherapy, pharmacological and social interventions were infrequently reported as recommended.

**Barriers to Implementation of DMD Care Considerations**

Across all disease management areas, clinic directors agreed that not all health providers are familiar with each Care Consideration (data not shown). Specifically, primary care and emergency/urgent care providers were reported as having less familiarity or understanding of the Care Considerations. Regarding system barriers, clinic directors agreed there is inadequate time at clinic visits to complete all recommendations, inadequate funding to support ancillary staff and inadequate coordination of care across disciplines and services. Of the potential patient or family barriers queried, all clinic directors agreed that distance to neuromuscular centers, out-of-pocket expenses and access to services/resources in the patient’s community were significant barriers to implementation of care. There was little consistency between clinic directors on whether there is a lack of awareness or familiarity with the content of the Care Considerations by patients and families.

**Discussion**

Directors of selected neuromuscular clinics located in the MD STARnet surveillance areas completed a pilot questionnaire describing their recommended clinical management of patients diagnosed with DMD. Their responses were compared to the Care Considerations published in 2010 to evaluate implementation across multiple disease management areas. Each director also provided a perspective about potential provider, system, and patient barriers to implementation of the Care Considerations. Overall, reported clinical practices related to the diagnostic process, access to a multi-disciplinary medical team, timeliness of follow-up by a medical professional specialized in the care of neuromuscular disorders, and recommendation of corticosteroids to all patients and monitoring their side effects were consistent with the Care Considerations and the recently released imperatives for DUCHENNE MD^15^ and the Transforming Duchenne Care Initiative.^12^ Rehabilitation interventions, such as stretching, orthotics for positioning, and standing devices were reported as recommended at various disease stages by all directors. In addition, implementation of cardiac monitoring and management showed high agreement with the Care Considerations for the use of echocardiograms in terms of initiation of studies, long-term follow-up studies, and referrals to a cardiac specialist. Finally, monitoring of orthopedic issues, specifically scoliosis, was consistent with recommended care.

There were several disease management areas for which broad adherence was observed, but variability in patterns of implementation were also evident. For example, although the frequency of respiratory function measurements for forced vital capacity and peak cough flow were consistent with the Care Considerations, other recommended measurements (e.g., blood gases, sleep studies, oxyhemoglobin saturation by pulse oximetry) were either not performed or only completed with signs and symptoms. A survey study of pediatric respirologists and Canadian Paediatric Neuromuscular Group (CPNG) members about respiratory consultations, frequency and reasons for assessing pulmonary function, and interventions showed similar findings.^7^ Responses were compared between provider types and showed differences in preferred tests for assessing pulmonary function. Like our data, the pediatric NMDs preferred cough peak flow, whereas pediatric respirologists preferred maximal inspiratory and expiratory pressures. Similarly, the use of non-invasive pulmonary interventions, such as cough assist and positive pressure, were reported as recommended by all directors in our study, but the initiation of use occurred under variable clinical signs. This variability may reflect, in part, the lack of empirical evidence for the optimal conditions under which non-invasive ventilation should be started.^7^^, ^16^, ^17 Management of bone health was another area that showed adherence with Care Considerations, but also demonstrated variability. The reported management of bone demineralization was consistent with recommendations, but the timing and frequency of monitoring bone health was not. Researchers have continued studying the use of DEXA scans to monitor bone health, which will inform on the accuracy with which it can be assessed and appropriate methods to do so in this patient population.^18^^, ^19

Research evaluating provider reports of adherence to the Care Considerations is limited. Prior to the release of the Care Considerations, but after the release of guidelines by the American Academy of Neurology, American Thoracic Society, and American Academy of Pediatrics, McMillan et al^6^ reported on findings from a questionnaire distributed to physicians who were members of the Canadian Pediatric Neuromuscular Group. Comparable to our findings, the reported clinical practice was largely consistent with recommendations from the existing guidelines. For those disease management areas that showed variability in adherence, the types of studies and levels of evidence associated with the recommendations were most often categorized as expert opinion with inadequate or conflicting directives given current knowledge (e.g., DEXA scans, respiratory assessments and interventions, cardiac assessments).^6^ Further, low rates of adherence for some interventions (e.g., pulmonary) in Canada were attributed to lack of coverage by insurance and balancing direct cost to families in the absence of efficacy data.^7^ Finally, the clinical management of neurocognitive and psychosocial issues showed poor adherence despite the high rates of these issues among patients diagnosed with DMD.^20^^, ^21^, ^22

Delays in clinical uptake of recommendations may be affected by multiple factors.^13^ Given the clinical complexity of DMD and required multidisciplinary interventions, barriers to comprehensive implementation of the recommended care practices may be intensified due to the need to disseminate and translate into practice across multiple disciplines. Further, barriers may differ by disease management areas. From our questions about barriers to care, most clinic directors reported that not all providers who manage patients with DMD are familiar with all areas of the Care Considerations, in particular, those providing primary care or treating DMD patients in the emergency room. Institutional limitations were found to be significant behavioral barriers due to inadequate space, time, and funding. Finally, access to treating facilities, community services/resources and out-of-pocket expenses were all viewed as significant barriers to provision of recommended care.

There are several limitations to the findings from this pilot questionnaire. This study relied on a non-random selection of clinic directors from medical facilities within the MD STARnet surveillance region. Because of the small sample size, we did not examine variability within clinics nor did we examine barriers as potential reasons for observed variability. Specialist practices were summarized by clinic directors and not provided by individual specialists who provided the care. Despite these limitations, this pilot study provides information on the clinical management of DMD patients several years following the release of the Care Considerations, but prior to the release of the updated considerations. In addition to reporting on adherence to specific recommendations, we described patterns of care as provided. This information is important for aligning current practice with further refinement of clinical recommendations. Finally, the questionnaire asked clinic directors about perceived barriers to implementing the Care Considerations, which may provide guidance on potential revisions to recommendations, promote scientific investigations to support the recommendations, and increase visibility of institutional challenges to providing the recommended care.

**Conclusion**

This study showed adherence to many of the assessments and interventions suggested in the Care Considerations. The areas showing less consistency could be classified as derived from expert opinion with little supporting data. Improvement in the implementation of all aspects of recommended care requires continued study into the health impact of the proposed recommendations and continued advocacy for coverage by agencies.

## Corresponding Author

Kristin Conway, PhD, Department of Epidemiology, College of Public Health, The University of Iowa, Iowa City, Iowa 52240, USA; Telephone: 319-335-4641 Email: kristin-caspers@uiowa.edu

## Data Availability Statement

Data is available upon request.

Deborah Fox, MPH, Director, Congenital Malformations Registry Chief, Birth Defects Registry and Surveillance Section (BDRS) New York State Department of Health, Empire State Plaza, Corning Tower, Rm 1203 Albany, NY 12237, Phone 518-402-7950, Fax 518-402-7959, deb.fox@health.ny.gov, www.health.ny.gov/birthdefects

## Ethics Statement

IRB approval was received by the New York State Department of Health Institutional Review Board (IRB number: 03-062/15-0643/15-0644). No identifiers were collected in the questionnaire. The New York State Department of Health de-identified the questionnaires and assigned random IDs for data entry by the Iowa collaborators. Participants were informed of the purpose of study, which is to inform on clinical practice and future care considerations, but posting of raw data was not disclosed.

## Competing Interest Statement

The authors have declared that no competing interests exist.
